# Systematic Review and Metanalysis of Oncomarkers in IPF Patients and Serial Changes of Oncomarkers in a Prospective Italian Real-Life Case Series

**DOI:** 10.3390/cancers13030539

**Published:** 2021-01-31

**Authors:** Miriana d’Alessandro, Laura Bergantini, Elena Torricelli, Paolo Cameli, Federico Lavorini, Maria Pieroni, Rosa Metella Refini, Piersante Sestini, Elena Bargagli

**Affiliations:** 1 Respiratory Diseases and Lung Transplantation, Department of Medical and Surgical Sciences & Neurosciences, Siena University Hospital, 53100 Siena, Italy; bergantini@student.unisi.it (L.B.); cameli3@student.unisi.it (P.C.); pieronim@unisi.it (M.P.); refini@unisi.it (R.M.R.); sestini@unisi.it (P.S.); bargagli2@unisi.it (E.B.); 2 Section of Respiratory Medicine, Department of Clinical and Experimental Medicine, University of Florence, 50134 Florence, Italy; elena.torricelli@uslcentro.toscana.it (E.T.); federico.lavorini@unifi.it (F.L.)

**Keywords:** idiopathic pulmonary fibrosis, oncomarker, lung cancer

## Abstract

**Simple Summary:**

This paper is a review of the literature on the clinical role of oncomarkers in idiopathic pulmonary fibrosis (IPF) progression, and a description of the routine oncomarker trend in IPF patients over the longest follow-up yet reported. This is the first meta-analysis to review the results of studies evaluating the predictive prognostic value of circulating oncomarkers (CEA, Ca15.3, Ca19.9, Ca125, and KL-6) for IPF. The study focused on the discovery of multiple biomarker signatures, such as combinations of oncomarkers, that are widely and routinely available in biochemistry laboratories. The combination of clinical parameters and biological markers could help achieve more accurate results regarding prognosis and response to treatment in IPF. Our results could pave the way for a more “personalized” medical approach to patients affected by IPF.

**Abstract:**

Background: Idiopathic pulmonary fibrosis (IPF) is a severe progressive interstitial lung disease. At 5-year follow-up, 15% of IPF patients develop lung cancer, which significantly reduces the survival rate. Here we review the literature on the clinical role of oncomarkers in IPF progression, and describe the trend of routine oncomarkers in IPF patients over the longest follow-up yet reported. Materials and methods: A systematic search of the literature in PubMed was performed to find relevant studies published up to 24 September 2020. The most common oncomarkers were chosen to select papers related to pulmonary fibrosis. Then, 24 IPF patients and 25 non-IPF patients, followed at Careggi ILD Referral Centre and Siena Regional Referral Centre for ILD, were enrolled consecutively. Results: A few studies reported an association between serum oncomarkers and severity of IPF. NSE, CEA, Ca19.9, and Ca125 were higher in the IPF, than in the non-IPF, group at every follow-up (*p* < 0.05). Ca15.3 concentrations were higher in the IPF, than the non-IPF, group at t3 (*p* = 0.0080) and t4 (*p* = 0.0168). To improve the specificity and sensitivity of Ca15.3, a panel of biomarkers was analyzed, with the IPF group as dependent variable, and chitotriosidase, Cyfra 21.1, Ca15.3, Ca125, and Ca19.9 as independent variables. Conclusions: This study focused on the discovery of multiple biomarker signatures, such as combinations of oncomarkers, that are widely and routinely available in biochemistry laboratories. The combination of clinical parameters and biological markers could help achieve more accurate results regarding prognosis and response to treatment in IPF. Our results could pave the way for a more “personalized” medical approach to patients affected by IPF.

## 1. Introduction

Idiopathic pulmonary fibrosis (IPF) is a chronic, progressive, fibrosing interstitial pneumonia of unknown cause, occurring primarily in older adults, and limited to the lungs [1]. Recent meta-analyses have shown close associations between the development of IPF and lung cancer [2,3,4]. Both usually affect the periphery of lower lung lobes, sharing common risk factors (e.g., smoking, environmental or occupational exposure, viral infections, and chronic tissue injury) and pathogenic mechanisms, such as epigenetic and genetic alterations, abnormal expression of microRNAs, cell and molecular aberrations (e.g., altered responses to regulatory signals, delayed apoptosis, and reduced cell-to-cell communication), and activation of specific signal transduction pathways [5]. In the PROFILE (prospective observation of fibrosis in the lung clinical endpoints) study, some of these oncomarkers, especially Ca19.9 and Ca125, were associated with increased mortality [6]. Very recently Balestro et al. corroborated the prognostic value of Ca19.9 in end-stage IPF [7]. Although several authors have reported high concentrations of common oncomarkers, including carcinoembryonic antigen (CEA) [8], cancer antigen 19.9 (Ca19.9) [9], 15.3 (Ca15.3) [10], 125 (Ca125) [8], and Krebs von den Lungen-6 (KL-6) [11,12,13,14], in IPF, little data is available on the prognostic role of all these markers, taken together, and their interactions, in IPF progression [9,15]. Here we review the literature on the clinical role of oncomarkers in IPF progression and describe the trend of routine oncomarkers in IPF patients over the longest follow-up yet reported.

## 2. Results 

### 2.1. Systematic Review and Metanalysis

#### 2.1.1. Search of the Literature

**Figure 1:**
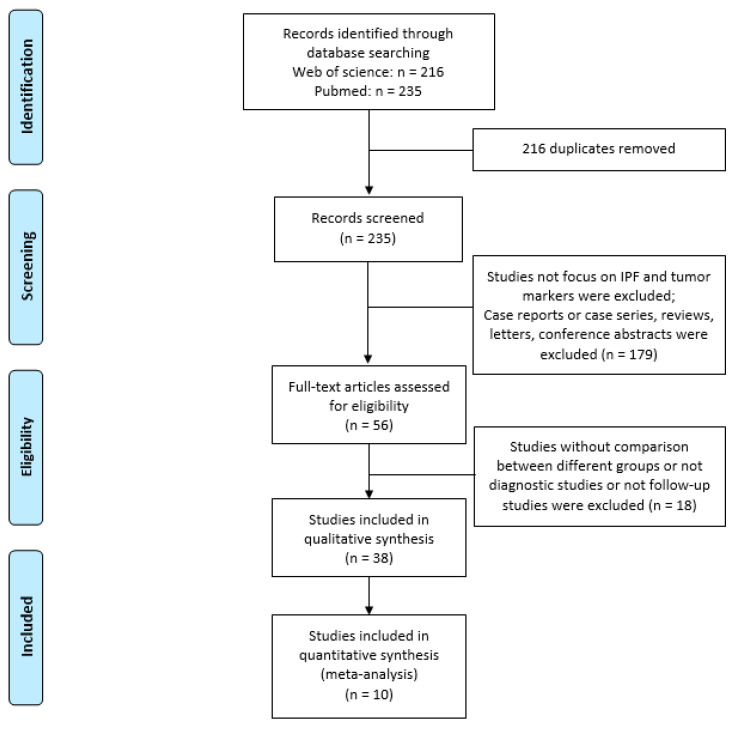
Flow diagram of chosen studies for the present metanalysis.

Figure 1 shows the flow diagram of the present systematic review. Mean concentrations and standard deviations (SD) of circulating oncomarkers were extracted from 10 studies for patients with and without IPF (IPF patients: n = 1757, non-IPF: n = 1508). Baseline circulating levels of serum oncomarkers of patients with and without IPF were obtained from all studies [9,10,15,16,17,18,19,20,21,22] to explore the predictive value for IPF progression (including development of lung cancer). Assessment of disease progression was performed according to the evaluation of pulmonary functional parameters (forced vital capacity (FVC) and/or diffusing capacity for carbon monoxide (DLCO)) and/or computed tomography (CT) features.

#### 2.1.2. Meta-Analysis Results

**Figure 2:**
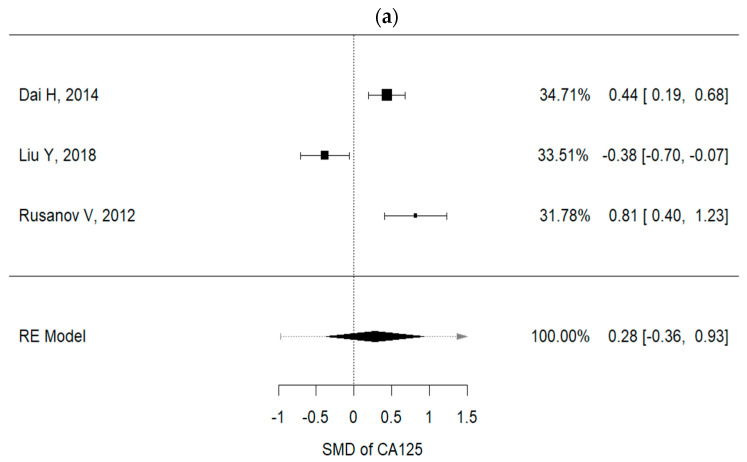

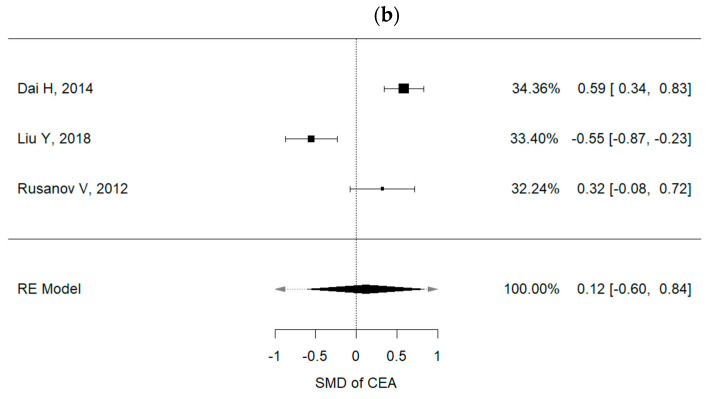
(**a**) Metanalysis results from Ca125 concentrations of selected studies; (**b**) metanalysis results from CEA concentrations of selected studies.

Higher concentrations of carcinoembryonic antigen (CEA) and Ca-125 were recorded in IPF patients with lung cancer, than in non-IPF patients (I^2^ = 92.3%, *p* < 0.001, mean CEA concentrations (IPF vs. non-IPF): 5.35 vs. 4.89 ng/mL; I^2^ = 91.9%, *p* < 0.001, mean Ca125 concentrations (IPF vs. non-IPF): 34.68 vs. 32.09 U/mL) (Figure 2a,b).

**Figure 3:**
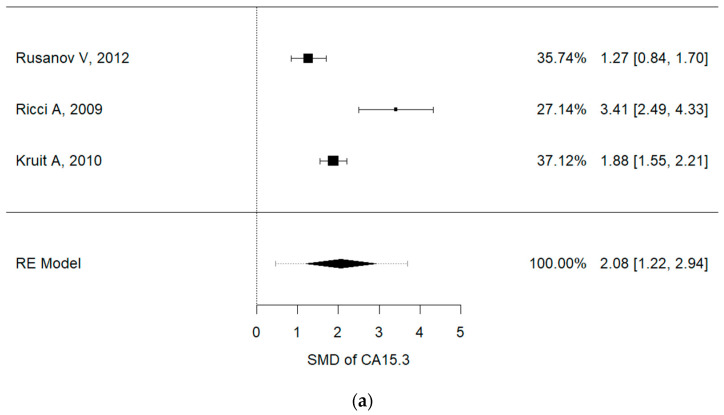

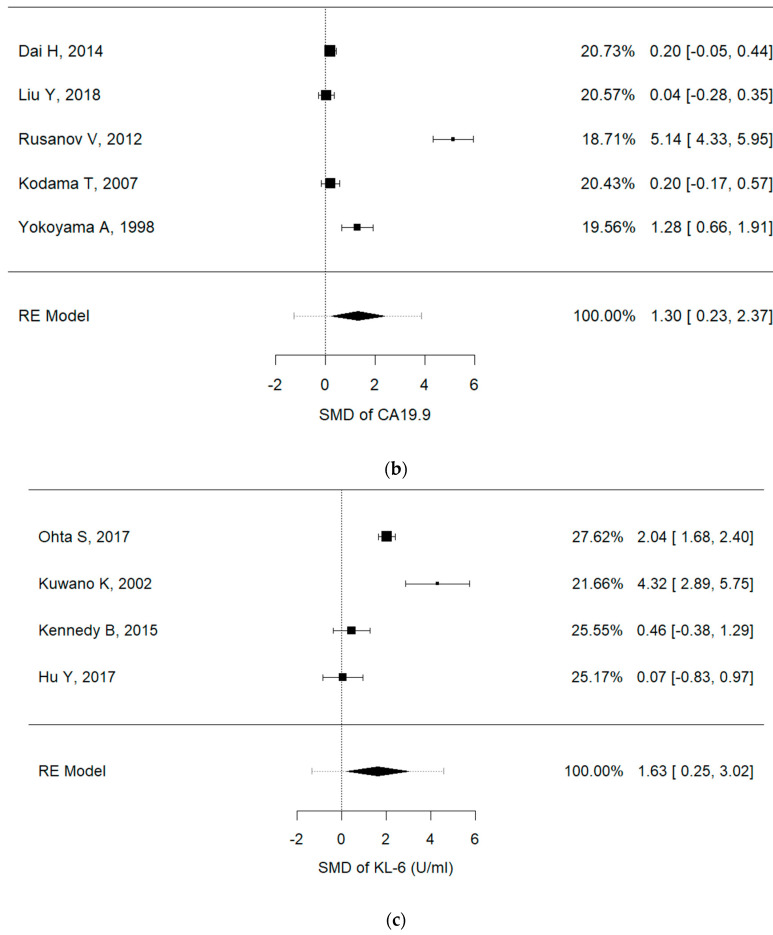
(**a**) Metanalysis results from Ca15.3 concentrations of selected studies, (**b**) metanalysis results from Ca19.9 concentrations of selected studies, (**c**) metanalysis results from KL-6 concentrations of selected studies.

Serum concentrations of Ca15.3 (I^2^ = 88.8%, *p* = 0.001, mean (IPF vs. non-IPF): 91.02 vs. 16.3 U/mL), Ca19-9 (I^2^ = 97.3%, *p* < 0.001, mean (IPF vs. non-IPF): 54.71 vs. 15.29 U/mL) and KL-6 (I^2^ = 91.9%, *p* < 0.001, mean (IPF vs. non-IPF): 1164 vs. 317 U/mL) were associated with disease progression in IPF patients (Figure 3a–c). In particular, higher values of these three markers were found in IPF patients and were correlated with a worse prognosis.

### 2.2. Original Contribution

#### Study Population

The main characteristics of our population are reported in Table 1. As expected, IPF patients were predominantly male (81.4%), over 65 years of age and most had a history of cigarette smoking (75%). Velcro crackles were audible by chest auscultation in all IPF patients, and significantly more often than in non-IPF patients (Table 1). Dyspnea expressed as modified Medical Research Council (mMRC) score was statistically different in the IPF and non-IPF groups at t3 (*p* = 0.0070). In the IPF group, mMRC score at t0 differed from those at subsequent follow-up times (*p* = 0.0001). At 18-month follow-up (t3), three IPF patients had died, while no patient had died in the non-IPF group. Stratifying the study population according to therapy with pirfenidone or nintedanib, we did not observe any statistically significant difference of oncomarker concentrations or functional disease progression.

Statistical analysis was performed comparing each sampling time for each group (IPF: t0 vs. t1, t0, vs. t2, etc.); moreover, a comparison analysis was performed between the two subgroups (IPF t0 vs. non-IPF t0, IPF t1 vs. non-IPF t1, etc.).

**Figure 4:**
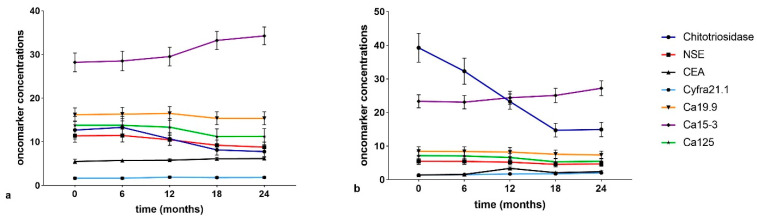
(**a**) Serial concentrations of oncomarkers in the IPF group. (**b**) Serial oncomarker concentrations in the non-IPF group.

Serum concentrations (Figure 4a,b) of chitotriosidase and oncomarkers Cyfra 21.1, Ca19.9, and Ca125 were in the normal range at t0 in the IPF and non-IPF groups. As expected, serum chitotriosidase was higher in the non-IPF group in relation to the presence of sarcoidosis patients (*p* < 0.05) [23,24,25,26,27]. This trend remained unchanged even at 18-month follow-up.

The non-IPF group showed lower CEA concentrations at t0 than at t3 (*p* = 0.0294) and t4 (*p* = 0.0019) and the difference was statistically significant between t1 and t4 (*p* = 0.0327). Comparing oncomarker concentrations in the two groups, neuron specific enolase (NSE), CEA, Ca19.9 and Ca125 were higher in IPF patients than in the non-IPF group at every follow-up (*p* < 0.05). Ca15.3 concentrations were higher in the IPF than the non-IPF group at t3 (*p* = 0.0080) and t4 (*p* = 0.0168).

In IPF group patients, serum concentrations of Ca15.3 showed a statistically significant increase in the intervals t0–t3 (*p* = 0.0369), t0–t4 (*p* = 0.0142), t1–t3 (*p* = 0.0350), and t2–t4 (*p* = 0.043).

CEA had the greatest sensitivity and specificity for distinguishing IPF and non-IPF patients at all follow-up times (Table 2).

**Figure 5:**
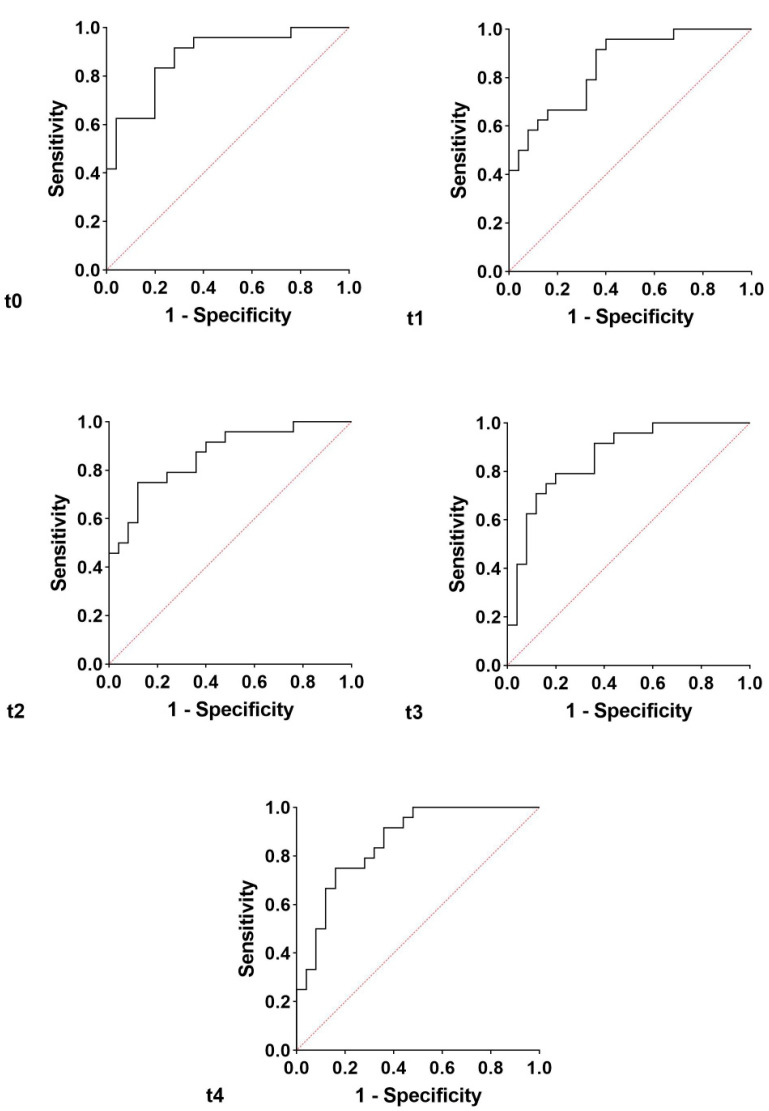
The analysis of logistic regression reporting t0, t1, t2, t3, and t4 oncomarkers panel in the IPF vs. the non-IPF group.

In order to improve the specificity and sensitivity of Ca15.3, a panel of biomarkers was analyzed. With the IPF group as dependent variable, and chitotriosidase, Cyfra 21.1, Ca15.3, Ca125, and Ca19.9 concentrations at t0 as independent variables, the area under the receiver operating curve (AUROC) obtained by logistic regression was 88% (95% CI 78–97, NPP 82.6%, and PPP 76.9%, *p* < 0.0001) (Figure 5). With the same biomarker concentrations at t1, t2, t3, and t4 as independent variables, we repeated the logistic regression. At t1, we obtained an AUROC of 85% (95% CI 74–95, NPP 70.8%, and PPP 68%, *p* < 0.0001) (Figure 5), at t2, 86% (95% CI 76–96, NPP 78.3%, and PPP 73.1%, *p* < 0.0001) (Figure 5), at t3, 86% (95% CI 76–96, NPP 80%, and PPP 79.2%, *p* < 0.0001) (Figure 5) and at t4, 86% (95% CI 75–96, NPP 78.3%, and PPP 73.1%, *p* < 0.0001) (Figure 5). With respect to a single biomarker, the panel increased sensitivity and specificity in discriminating the two groups at all follow-up times.

**Figure 6:**
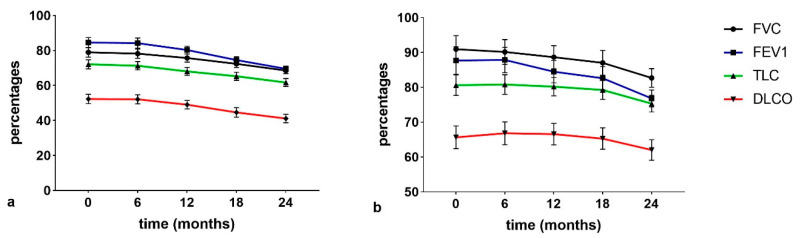
(**a**) IPF serial changes of pulmonary function test (PFT) parameters (**b**) non-IPF serial changes PFT parameters.

Regarding lung function (Figure 6), FVC%, forced expiratory volume in 1 s (FEV1%), total lung capacity (TLC)%, and DLCO% decreased significantly in the interval t0–t4 in IPF patients compared to non IPF patients. TLC and DLCO percentages were lower in the IPF than in the non IPF group at all follow-ups. In IPF patients, all functional parameters were significantly different (*p* < 0.01) at t3 with respect to t2 and t0 (*p* < 0.01). No significant differences (*p >* 0.01) in lung function parameters were observed in non-IPF patients in the serial follow-up.

The trend of functional parameters in the IPF population showed a progressive statistically significant decline at t3 and t4 (*p* < 0.05). Due to the limited statistical sample, no correlations between serological biomarkers and survival data could be detected. Correlation analysis between serum biomarkers and lung function parameters in the two groups are shown in Table 3. Interestingly, there was a significant negative correlation between serum concentrations of CEA and FEV1, FVC and DLCO percentages at t3 and t4.

## 3. Discussion

This is the first meta-analysis to review the results of studies evaluating the predictive prognostic value of circulating oncomarkers (CEA, Ca15.3, Ca19.9, Ca125, and KL-6) for IPF. Although oncomarker concentrations were higher in IPF than non-IPF patients, no data were available on IPF progression, including mortality. A few studies have found higher levels of circulating oncomarkers in IPF than in non-IPF patients [8,9,10,15,16,18,22]. The present study showed that higher circulating levels of CEA, Ca15.3, Ca19.9, and Ca125 in IPF patients than in non-IPF patients may be due to the common molecular pathways shared by IPF and lung cancer. Moreover, increased KL-6 production may be due to regenerating type II alveolar epithelial cells, and/or increased permeability caused by damage to the air–blood barrier in interstitial lung disease (ILD) [14].

A few studies have reported an association between serum oncomarkers and severity of IPF, and only two papers have evaluated the association with survival of these patients. These papers reported that elevated CEA and Ca125 concentrations were associated with increased risk of lung cancer in IPF patients with similar cut-off values [8,16]. Kodama T et al. suggested that clinicians pay attention to evidence that elevated serum levels of CA19.9 may be related to poor prognosis in IPF patients [9]. Balestro et al. recently reported CA 19-9 as a disease severity marker in patients with end-stage ILD, demonstrating an inverse correlation of this oncomarker with functional decline, particularly among patients with rapidly progressive IPF [7]. Ca15.3 has been studied in relation to the pathogenesis of IPF, and it has been associated with survival and disease severity, due to an inverse correlation between DLCO and high-resolution computed tomography (HRCT) findings [15].

One study showed a significant negative correlation of serum KL-6 levels with FEV1 and FVC percentage [21]. The present study also found that circulating KL-6 levels were highly valuable in the prognosis of IPF [20], indicating a significant association between the baseline levels of circulating KL-6 and mortality in IPF.

In the present study, we evaluated serial changes in chitotriosidase and circulating oncomarkers in a cohort of ILD patients divided into IPF and non-IPF groups. Increased chitotriosidase values have been repeatedly reported in sarcoidosis patients, and may predict clinical course and potential relapse of the disease [23,24,25,26,27,28]. As expected, serum chitotriosidase in our non-IPF group was high in relation to the presence of sarcoidosis patients. Moreover, significant direct correlations between chitotriosidase and functional parameters were observed in the non-IPF group at baseline, and overtime, confirming the role of this protein as a potential prognostic marker.

The clinical course of IPF is variable, ranging from slow progression over many years, to acute exacerbation and rapid loss of lung function. This, associated with a lack of biomarkers to predict disease progression and response to treatment, makes the clinical management of IPF very challenging [29,30,31,32,33,34,35]. Thus, interest has been focused on the discovery of multiple biomarker signatures that could be used more effectively in the diagnosis and prognosis of IPF. In 2016, White and co-workers developed a panel of 35 extracellular matrix, extracellular matrix-related, and lung-specific analytes, measured in the plasma of IPF patients, to create a diagnostic score [36]. Other studies have shown increased prognostic accuracy when serum biomarkers were used in combination [37,38]. The combining of clinical parameters and biological markers has been studied in order to achieve more accurate results regarding the prognosis of IPF [12,36]. In this context, the present study identified and validated new biomarkers of IPF for their prognostic potential. Serial concentrations of NSE, CEA, Ca19.9, and Ca125 were higher in IPF than in non-IPF patients at each follow-up. In particular, Ca15.3 concentrations were higher in the IPF than in the non-IPF group at t3 and t4, and showed an increasing trend.

Significant correlations between oncomarkers and several functional parameters were found. In particular, CEA showed an indirect correlation with FEV1%, FVC%, and DLCO% decline at 18- and 24-month follow-up in IPF patients with respect to non-IPF patients. Our findings were in line with those of a prospective study by Fahim et al., who demonstrated elevated serum concentrations of CEA in IPF patients, and a significant negative correlation of the latter with lung function parameters [39]. Moreover, a mechanism of elevation of this tumor antigen is suggested by immunohistochemical evidence of CEA staining of metaplastic alveolar epithelium lining honeycomb cysts and respiratory bronchioles [39]. Although the exact mechanism is unknown, the atypical epithelial proliferation and squamous metaplasia seen in lung biopsies of patients with idiopathic interstitial fibrosis may be one of the mechanisms responsible for increased CEA in IPF. In addition to elevation of CEA in pulmonary fibrosis of unknown etiology, there is evidence of significantly elevated levels of cancer antigen Ca15.3 in IPF and advanced sarcoidosis [10,40]. Fujita and colleagues showed intense staining of Ca15.3 in fibroblasts of fibroblastic foci and lung fibroblast cultures from IPF patients [41].

Our results showed that CEA had the greatest sensitivity and specificity in distinguishing IPF from non-IPF at all follow-up times. Ca15.3 had a low sensitivity, but was the only marker to show a rising trend in IPF. However, a panel consisting of chitotriosidase, Cyfra 21.1, Ca15.3, Ca125, and Ca19.9 showed better sensitivity and specificity at all follow-up times than any single biomarker in distinguishing IPF from non-IPF patients. Histological assessment of Ca19.9 and Ca125 suggested that these proteins were markers of epithelial damage [40]. In the PROFILE study, these biomarkers were both associated with high mortality [6]. If these findings are corroborated in future studies, Ca125 and Ca19.9 could become point-of-care prognostic markers.

Our data confirms the previous findings of higher serum levels of oncomarkers (Ca125, Ca15.3, Ca19.9, and CEA) in IPF than in non-IPF patients. Ca15.3 was correlated with poor prognosis. Interestingly, all except CEA decreased significantly after lung transplant [9]. It would be worthwhile validating these interesting results in multicentric prospective studies with a larger sample of patients. It would also be interesting to measure oncomarker concentrations in other biological materials.

The limitations of the present study were the relatively small number of studies found for the meta-analysis, which did not enable us to explore the sources of heterogeneities.

## 4. Materials and Methods

### 4.1. Systematic Review and Metanalysis

#### 4.1.1. Search of the Literature

A systematic search of the literature in PubMed was performed to find relevant studies published before 24 September 2020, and limited to the following publication types (clinical study, journal article, multicenter study, observational study), species (human), and languages (English). The most common oncomarkers (Ca15.3, Ca19.9, Ca125, alpha-fetoprotein, prostate-specific antigen, calcitonin, chromogranin A, neuron-specific enolase, Cyfra 21-1, human chorionic gonadotropin, S100, thyroglobulin, and KL-6) were chosen in order to select papers related to pulmonary fibrosis. The following medical subject headings were searched in (Title/Abstract): (“Ca-15-3” or “Ca15.3” “pulmonary fibrosis”), (“Ca19.9” “pulmonary fibrosis”), (“Ca125” “pulmonary fibrosis”), (“CEA” “pulmonary fibrosis”), (“alpha-fetoprotein” “pulmonary fibrosis”), (“prostate specific antigen” “pulmonary fibrosis”), (“calcitonin” “pulmonary fibrosis”), (“chromogranin A” “pulmonary fibrosis”), (“neuron specific enolase” or “NSE” “pulmonary fibrosis”), (“Cyfra 21-1” “pulmonary fibrosis”), (“human chorionic gonadotropin” “pulmonary fibrosis”), (“S100” or “calgranulin” “pulmonary fibrosis”), (“thyroglobulin” “pulmonary fibrosis”), (“Krebs von den Lungen-6” or “KL-6” or “MUC1” “pulmonary fibrosis”).

#### 4.1.2. Study Selection

Studies were included in the meta-analysis when they met the following criteria: studies focused on patients diagnosed with IPF according to the results of chest radiography, conventional thoracic computed tomography, HRCT, and pulmonary function tests (either FVC% or DLCO% < 80%); oncomarkers identified in serum; homogeneous units of measurement and methods of detection of oncomarkers; studies that provided sufficient data to complete cross-tabulations (2 × 2 tables) for evaluating the diagnostic accuracy of serum oncomarkers in IPF versus non-IPF patients. When the same population was published in different reports, the most recent or complete report was included. Case reports, case series, reviews, letters and conference abstracts were excluded due to limited assessment or analysis of data. Studies with insufficient data (or that only reported the cut-off value) for completing cross-tabulations were excluded as well.

#### 4.1.3. Data Collection and Meta-Analysis

Data was extracted from eligible studies and summarized independently by two researchers (MD and LB). Any disagreement was resolved by consensus. The following information was collected from each study: first author’s name, year of publication, disease subtypes, affiliation of study, ethnicity of population, assay method, cut-off value, sample size, number of case groups, number of control groups. From cross-sectional studies we extracted mean concentrations and standard deviations (SD) of circulating oncomarkers in patients with and without IPF.

These data were processed with Covidence, Jamovi, and GraphPad Prism 9 software. For cross-sectional studies, the standard mean difference (SMD) and 95% CI were computed as the effect size of following comparisons: IPF vs. non-IPF. Heterogeneity between studies and the amount of variation derived from heterogeneity were evaluated by Q test and I^2^, respectively. When heterogeneity was high (*p* value for Q test ≤ 0.05, I^2^ ≥ 50%), random effects models were used as a pooling method.

### 4.2. Original Contribution

#### 4.2.1. Study Population

Seventy-nine ILD patients followed at Careggi Interstitial Lung Diseases Referral Centre and Siena Regional Referral Centre for ILD were enrolled consecutively. Twenty-four diagnosed with idiopathic pulmonary fibrosis (IPF) and 25 non-IPF patients diagnosed with nonspecific interstitial pneumonia (NSIP) (n = 9), sarcoidosis (n = 8), pleuroparenchymal fibroelastosis (PPFE) (n = 2), respiratory bronchiolitis associated with ILD (RB-ILD), subacute hypersensitivity pneumonitis (HP) (n = 4), and cryptogenic organizing pneumonia (COP) (n = 2) were selected for consecutive serial evaluation of oncomarkers. We excluded IPF patients with a follow-up inferior to 24 months, those patients with concomitant infection, malignancy and acute exacerbation compliance, or those patients with non-definite usual interstitial pneumonia (UIP) pattern at CT scan. All the diagnoses were confirmed by multidisciplinary discussion, according to international guidelines (ATS/ERS).

Of this selected population, 15 IPF patients (62.5%) were treated with pirfenidone and nine (37.5%) with nintedanib. Non-IPF patients were treated with corticosteroids (n = 16, 64%) and immunosuppressant therapy (azathioprine, mycophenolate ecc descrivere) (n = 9, 36%). All patients were naïve for therapies at the time of diagnosis (t0), and started nintedanib or pirfenidone treatments according to Italian national drug inclusion criteria.

Medical history, physical examination, age, sex, ethnicity, BMI, vital signs, onset of symptoms, hospitalizations, concomitant diseases, smoking history, occupational history, current and previous therapy, blood gas analysis, and 6-minute walking test were recorded in a database. All patients gave written informed consent to participation in the study, which was approved by the local ethics committee CEAVSE (code number 180712; Markerlung 17431).

#### 4.2.2. Methods

Lung function tests were performed according to ATS/ERS recommendations using a plethysmograph with corrections for temperature and barometric pressure and the parameters were expressed as % of predicted value: forced expiratory volume in 1 s (FEV1%), (FVC%), (DLCO%) by the single-breath method, and total lung capacity (TLC%). A six-minute walking test was performed according to international recommendations. These measurements were obtained from all patients able to perform lung function tests.

Serum samples for assay of the following proteins/oncomarkers were drawn at baseline t0 and every 6 months of follow-up (6 months (t1), 12 months (t2), 18 months (t3), and 24 months (t4)): chitotriosidase (chitinase-1), carcinoembryonic antigen (CEA), cancer antigen 15-5 (Ca15-3), neuron-specific enolase (NSE), cancer antigen 19-9 (Ca19-9), cytokeratin fragment 21-1 (Cyfra 21.1), and cancer antigen 125 (Ca-125). Oncomarkers and chitotriosidase were assayed as previously reported [7,8,10,23,39,42,43].

#### 4.2.3. Statistical Analysis

Data was expressed as mean ± standard deviation or median and interquartile range. Non-parametrical tests were adopted for data analysis: Kruskal–Wallis and Dunn´s multiple tests to compare the two groups (IPF and non-IPF) and to compare the sampling times each other (t0, t1, t2, t3, t4). The Chi-squared test was used for categorical variables, as appropriate. The oncomarkers analyzed in this study were commonly used in clinical practice and the cut-off values were standardized: CEA > 2.5 ng/mL (normal value 0 to 2.5–3 ng/mL), Ca15-3 > 35 UI/mL (n.v. 0 e 32.4 U/mL), NSE > 15 (n.v. 0 to 15 ng/mL), Ca19-9 > 40 U/mL (n.v. 0–40 U/mL), Cyfra 21.1 > 3.5 mcg/mL (n.v. 0 to 3.5 mcg/mL), and Ca125 > 35 UI/mL (n.v. 0–35 UI/mL).

Serum biomarker concentrations were also compared between groups, assessing areas under (AUC) the receiver operating characteristic curves (ROC). Logistic regression analysis, using the IPF group as dependent variable against non-IPF patients, to assess the potential of serum markers in discriminating the two groups at each sampling time, was adopted. Sensitivity, specificity, and positive and negative predicted values (PPV and NPV, respectively) were calculated for cut-off of the different variables. The Spearman test was used to look for correlations between variables. A p value less than 0.05 was considered statistically significant. All the statistical analysis and the related figures were carried out using GraphPad Prism 8.4 software.

## 5. Conclusions

In conclusion, the study focused on the discovery of multiple biomarker signatures, such as combinations of oncomarkers, that are widely and routinely available in biochemistry laboratories. The combination of clinical parameters and biological markers could help achieve more accurate results regarding prognosis and response to treatment in IPF. Our results could pave the way for a more “personalized” medical approach to patients affected by IPF.

Biomarkers are relevant in medicine, particularly in the realm of “personalized medicine”. Disease severity biomarkers are helpful tools, and they are valuable for predicting prognosis. It is known that every patient can have a different response to a specific therapeutic approach according to several immunological features. Our results could pave the way for a more “personalized” medical approach to patients affected by IPF monitoring oncobiomarkers.

## Figures and Tables

**Table 1:** The main characteristics of our population divided in idiopathic pulmonary fibrosis (IPF) and non-IPF groups. 1 and 2: older and prevalence of males in the IPF group (*p* < 0.05), respectively. 3. Velcro sound was prevalent in the IPF group (*p* < 0.05). Abbreviations: modified Medical Research Council (mMRC).

	IPF (n = 24)	Non-IPF (n = 25)
Age (yr)	73.80 ± 7.79 ^1^	62.43 ± 13.63
Sex (M/F)	22/2 ^2^	14/11
Smoking history (>5 p/yr)	18/24	12/25
Familiarity for ILD (yes/no)	2/24	1/25
Cough (VAS > 3/10 cm)	22/24	17/25
Dyspnea (mMRC > 1/4)	16/24	14/25
Velcro sound (yes/no)	23/24 ^3^	8/25
Clubbing (yes/no)	3/24	1/25

**Table 2:** Receiver operating characteristic (ROC) curve analysis between IPF and non-IPF patients according to oncomarker concentrations at each sampling time. Abbreviations: t0, baseline; t1, 6 months; t2, 12 months; t3, 18 months; t4, 24 months.

IPF vs. Non-IPF	AUC	*p* Value	Cut-Off Value	Sensitivity	Specificity
NSE, t0	76	0.0016	7.75	72	70.8
NSE, t1	77.1	0.0012	4.96	68	79.2
NSE, t2	76.7	0.0014	7.95	72	66.7
NSE, t3	77.4	0.0010	4.65	72	75
NSE, t4	71.6	0.0096	5	72	70.8
CEA, t0	94	<0.0001	2.55	96	87.5
CEA, t1	99.7	<0.0001	2.85	96	95.8
CEA, t2	95.6	<0.0001	2.85	92	95.5
CEA, t3	98.8	<0.0001	2.85	88	95.8
CEA, t4	98.1	<0.0001	3.3	88	95.8
Ca19.9, t0	78.4	0.0006	12.6	76	79.2
Ca19.9, t1	77	0.0012	11.9	72	79.2
Ca19.9, t2	78.2	0.0007	11.2	68	79.2
Ca19.9, t3	79.6	0.0004	9.7	68	79.2
Ca19.9, t4	81.6	0.0002	8.3	68	83.3
Ca15-3, t3	69.9	0.0168	31.6	72	54.2
Ca15-3, t4	67.7	0.0340	29.3	60	62.5
Ca125, t0	71.4	0.0114	8.8	68	66.7
Ca125, t1	72.1	0.0008	8.7	68	66.7
Ca125, t2	74.2	0.0036	9.3	68	66.7
Ca125, t3	73.8	0.0042	7.2	72	66.7
Ca125, t4	71.5	0.0099	9.5	76	62.5

**Table 3:** Correlation analysis between serum biomarkers and pulmonary function test (PFT) parameters in the two subgroups. Abbreviations: FVC, forced vital capacity; FEV1, forced expiratory volume in 1 second; DLCO, diffusing lung for carbon monoxide; CEA, carcinoembryonic antigen; TLC, total ling capacity.

IPF		Rho Coefficient	*p* Value	Non-IPF		Rho Coefficient	*p* Value
t0				t0			
CEA	TLC	−0.48	0.018	Chito	FVC	0.476	0.016
Cyfra21.1	DLCO	−0.43	0.036		FEV1	0.425	0.034
t1					TLC	0.422	0.035
Chito	DLCO	0.511	0.011	t1			
Cyfra21.1	DLCO	−0.44	0.031	Chito	FVC	0.605	0.001
t2					FEV1	0.662	0.0003
CEA	FVC	−0.501	0.013		TLC	0.515	0.008
CEA	FEV1	−0.524	0.009		DLCO	0.490	0.013
Cyfra21.1	FEV1	−0.430	0.036	t2			
t3				Chito	FVC	0.636	0.0006
Chito	FVC	0.416	0.043		FEV1	0.710	0.0001
Chito	FEV1	0.429	0.037		TLC	0.522	0.0075
CEA	FVC	−0.682	0.0002		DLCO	0.477	0.0252
CEA	FEV1	−0.811	0.000001	NSE	TLC	−0.480	0.015
CEA	DLCO	−0.647	0.001	CEA	FEV1	−0.529	0.007
Ca19.9	FVC	−0.458	0.024		TLC	−0.418	0.038
Ca15-3	FEV1	−0.439	0.032		DLCO	−0.423	0.035
t4				Ca19.9	TLC	−0.411	0.041
Chito	FEV1	0.54	0.006	t3			
CEA	FVC	−0.803	0.000002	Chito	FVC	0.635	0.0006
CEA	FEV1	−0.852	0.0000001		FEV1	0.783	0.000004
CEA	TLC	−0.464	0.022		TLC	0.579	0.002
CEA	DLCO	−0.520	0.009		DLCO	0.509	0.0093
Cyfra21.1	FVC	−0.427	0.037	NSE	TLC	−0.510	0.009
	FEV1	−0.505	0.012		DLCO	−0.410	0.0417
				CEA	TLC	−0.461	0.02
				Ca19.9	TLC	−0.461	0.0387
				Ca125	TLC	−0.408	0.0431
				t4			
				Chito	FVC	0.614	0.001
					FEV1	0.711	0.00007
					TLC	0.598	0.002
					DLCO	0.485	0.014
				NSE	TLC	−0.431	0.032
				Ca19.9	TLC	−0.419	0.037
				Ca125	TLC	−0.405	0.045

Abbreviations: t0, baseline; t1, 6 months; t2, 12 months; t3, 18 months; t4, 24 months.
